# Visuospatial impairment in dementia: a new index to improve the clinical diagnosis of Alzheimer’s disease

**DOI:** 10.1007/s40520-025-03028-1

**Published:** 2025-04-18

**Authors:** Francesca Santagata, Stefano F. Cappa, Roberto Presta, Caterina Burgio, Chiara Luppi, Massimiliano Massaia, Elisa Calvi, Patrizia D’Amelio

**Affiliations:** 1 Department of Medical Sciences, University of Turin, Turin, Italy; 2 University Institute for Advanced Studies IUSS, Pavia, Italy; 3 IRCCS National Neurological Institute Mondino Foundation, Pavia, Italy; 4 Geriatric Medicine and Geriatric Rehabilitation Unit, Lausanne University Hospital (CHUV), Lausanne, Switzerland; 5 Center for Cognitive Disorders and Dementia, Section of Geriatrics, City of Health and Science University Hospital, Turin, Italy

**Keywords:** Dementia, Alzheimer’s disease, Neuropsychological assessment, Visuospatial function

## Abstract

**Background:**

The differential diagnosis between Alzheimer’s disease (AD) and other causes of dementia is essential but challenging. Therefore, there is an increasing need for early, reliable, and non-invasive tests to distinguish between different forms of dementia.

**Aims:**

To determine whether neuropsychological tests assessing visuospatial function can improve confidence in the clinical diagnosis of AD.

**Methods:**

Retrospective observational single-center cohort study involving all patients consecutively referred to our outpatient clinic for cognitive disorders who underwent neuropsychological assessment between 2013 and 2018. In addition to demographic and functional variables, each patient underwent neuropsychological tests to assess cognitive performance, memory, and executive, language, and visuospatial ability, according to clinical protocols. The clinical diagnosis of cognitive disorders, based on standard diagnostic criteria, served as the gold standard. Accuracy measures of visuospatial tests to diagnose AD were calculated. Additionally, a new index derived from the sum of four items (Rey-Osterrieth figure copying, Copy of Drawings, Clock Drawing Test, and years of schooling) was tested (ReDCOOL).

**Results:**

Of the 342 patients analyzed, 308 were diagnosed with dementia or mild cognitive impairment, including 60 with AD. AD patients exhibited the worst performance in visuospatial tests, and the utilization of the ReDCOOL index proved to be more dependable in identifying AD compared to other tests (AUROC 0.729, 95%CI 0.659–0.799; *p* < 0.001).

**Conclusion:**

The ReDCOOL index appears to increase confidence in the clinical diagnosis of AD compared to each of the visuospatial tests considered. Furthermore, this index is easily calculated and does not prolong the time needed for clinical evaluation, as it does not require a customized patient assessment.

**Supplementary Information:**

The online version contains supplementary material available at 10.1007/s40520-025-03028-1.

## Introduction

The aging population is steadily increasing, with projections suggesting that by 2050, over 1.6 billion individuals worldwide will be aged 65 and older [1]. In this aging society, a significant challenge for the future will be effectively addressing the rise in chronic diseases such as cognitive impairment. According to the WHO, approximately 55 million people worldwide were living with dementia in 2019, with projections indicating this number could rise to 139 million by 2050 [2]. About two-thirds of these cases are attributed to Alzheimer’s disease (AD), while others are diagnosed with vascular dementia, mixed dementia, or Lewy body dementia (LBD). The need for early, reliable, and cost-effective non-invasive tests to differentiate between various forms of dementia continues to grow.

Biomarkers measured in cerebrospinal fluid or through amyloid PET imaging remain underutilized and largely restricted to research centers. Consequently, clinical diagnosis remains the primary method, leading to delays in treatment and impeding research progress [3]. Tailored neuropsychological assessments provide a cost-effective approach to improving diagnostic accuracy in the identification of AD patients. However, many deficits observed in AD, such as episodic memory impairment, are not exclusive to this disease but also present in other forms of dementia, such as frontotemporal dementia (FTD) [4, 5]. Similarly, language deficits are not specific to AD [6], as they result from declines in semantic and pragmatic levels of language processing, seen in various other neurodegenerative conditions [7].

Memory and language impairments stem from brain damage affecting frontal and temporal lobes, regions often implicated in neurodegenerative diseases beyond AD. As a result, neuropsychological tests assessing these domains show poor specificity [8–10]. In contrast, visuospatial function is supported by large scale brain networks involving multiple brain regions, with the parietal lobe [11] acting as a central hub [12]. Actually, the parietal lobe’s impairment is an early and prominent characteristic of AD [13–15]. Although visuospatial processing also engages other brain areas, including the occipital, temporal, and frontal cortices, and subcortical structures, these areas may also be affected in AD, contributing to the observed visuospatial deficits.

Drawing abilities, which require a combination of skills such as object perception, image construction, and planning, are particularly useful in assessing visuospatial function. Drawing disorders, such as simplifications, spatial distortions, perseverations, and planning errors, can be identified through commonly used neuropsychological tasks. These impairments may be influenced by patient characteristics such as educational level, age, and culture, as well as the specific test used [16] and may provide valuable clinical insights for the differential diagnosis of various forms of dementia [17]. Impairments in visuospatial or planning abilities result in “constructional apraxia”, which is associated with brain lesions in the parietal and frontal regions [18]. While constructional impairment is commonly observed in different types of dementia, it may serve as an early and relatively specific marker of AD [19].

By examining visuospatial function in a large retrospective cohort, our study aims to capture the broader network dysfunction associated with AD. We hypothesize that patients with AD will exhibit distinct visuospatial impairments, particularly in tasks requiring spatial organization and construction, due to early involvement of the parietal lobe. Additionally, we propose that integrating multiple drawing tests into a novel scoring system will improve diagnostic accuracy by capturing a broader range of visuospatial deficits, aiding in the differentiation between AD and other dementias, such as frontotemporal dementia, where visuospatial function is less severely affected in the early stages. This approach may serve as a simple, non-invasive, and cost-effective screening tool to help identify candidates for further diagnostic evaluation using disease biomarkers [20].

## Materials and methods

This retrospective observational single-center cohort study was conducted at the “City of Health and Science” University Hospital in Turin, northern Italy. The study enrolled all patients consecutively referred to our outpatient clinic for cognitive disorders who underwent a neuropsychological assessment between January 2013 and December 2018.

The study adhered to the Recommendations Guiding Physicians in Biomedical Research Involving Human Subjects, received approval from the local Ethics Committee, and was reported in accordance with the Standards for Reporting Diagnostic accuracy studies (STARD) guidelines (STARD checklist available in Supplementary Material) [21].

### Descriptive variables and outcome

Data were retrospectively collected from medical records using standardized evaluation protocols by senior specialists in Geriatrics and neuropsychologists.

Besides demographic characteristics (i.e., age, sex, and years of education), functional status was evaluated utilizing Katz’s Basic Activities of Daily Living (BADL), which examines the requirement for assistance in tasks such as bathing, dressing, toileting, transferring, continence, and feeding (range 0–6, with 0 indicating complete functional dependency) [22]. Additionally, a modified version of the Instrumental Activities of Daily Living scale (IADL) was employed to assess autonomy in activities such as using the telephone, public transportation, managing medications and finances, and shopping (range 0–14, with scores below 10 indicating partial or complete lack of autonomy) [23]. The Disability Assessment for Dementia scale (DAD) was administered to the patients’ caregivers [24]. The DAD is a 40-item questionnaire specifically designed to evaluate functional disability in individuals with dementia: lower scores are indicative of poorer functional status [24]. Patients with known primary visual defects or impairments that prevented them from performing the drawing tests were excluded from the study.

### Cognitive assessment

According to clinical protocols, all patients were initially evaluated using the Italian version of the Mini-Mental State Examination (MMSE) [25]. For patients with an MMSE score higher than 24, the Italian version of Montreal Cognitive Assessment test (MoCA) was administered [26].

In addition to MMSE and MoCA, all the patients underwent a neuropsychological assessment battery to evaluate memory, executive/attentional functioning, language skills, visuospatial and constructional praxis all the tests applied were standardized and adjusted for age and education level. All patients were clinically diagnosed with one of the following disorders based on current criteria: AD [27], different variants of FTD [6], LBD [28], vascular dementia [29], mixed dementia (i.e., AD and vascular dementia) [30], or mild cognitive impairment (i.e., selective cognitive disturbances without functional impairment; MCI) [31]. The diagnosis of MCI was based on core clinical criteria and does not allow the identification of MCI due to AD [31]. Patients without any cognitive impairment were classified as having subjective cognitive decline (SCD).

### Neuropsychological assessment

A standardized neuropsychological assessment was performed in all patients to evaluate short-term and long-term memory, attention, executive functions, and language (tests adopted are reported in Table 1a).

**Table 1:** (a) Neuropsychological assessment and tests adopted

Domain	Sub-domain	Test
Short-term memory	Visuospatial	Corsi block-tapping test [33, 45]
	Verbal	Digit span [33, 45] Word disyllabic and digit span tests [33, 45]
Long-term memory	Visuospatial	Rey-Osterrieth Complex Figure (ROCF) [32]
	Verbal	Babcock Story Recall Test [33] Immediate and delayed recall of Rey’s 15 words [46]
Attentive function	Selective and sustained attention	Attentive Matrices [33]
	Divided attention, visual-motor coordination, mental flexibility, and conceptual shifting	Trail Making Test (TMT) [47]
Executive function	Conceptualization, mental flexibility, motor programming, sensitivity to interference, inhibitory control, and environmental autonomy	Frontal Assessment Battery (FAB) [48]
Language	Phonemic	Phonemic fluency test [33, 49]
	Semantic	Category fluency tests [33, 49]
	Verbal comprehension	Token Test (TT) [33]

**Table 1:** (b) Index test adopted for the assessment of visuospatial function and constructive Apraxia

Test	No. of items	Points per item	Total score (min-max)	Cut-off
ROCF copying test [32]	18	0.5: absent or unrecognizable reproduction 1: distorted, incomplete but placed properly, or complete but placed poorly reproduction 2: correct reproduction	9–36	< 28.88
Copy of Drawings (CD) [33]	7	0: unrecognizable, closing-in behavior 1: partially defective but recognizable copy 2: perfect copy	0–14	≤ 8
Free-drawn Clock Drawing Test (CDT) [34]	15	0: absent 1: present	0–15	< 7.57

The assessment of visuospatial function and constructive apraxia involved three index tests: (i) the ROCF copying test [32]; (ii) the Copy of Drawings (CD) [33]; (iii) the free-drawn Clock Drawing Test (CDT) [34]. Further details and adopted thresholds for positive test are reported in Table 1b.

Moreover, patients with dementia, when presented with drawing tasks, often exhibit a tendency to copy the proposed figure near to or overlapping the original model. This behavior, referred to as “closing-in behavior” (CIB), is often associated with frontal dysfunction, but is also common in AD [35] and has been linked to parietal damage [36]. In the present study, CIB was assessed based on performance in the copy of drawings [33] and in the ROCF copying tests [32]. CIB was evaluated by trained personnel and categorized as follows: (i) CIB Overlap Type, if part of the copy encroaches upon the model space in the upper half of the sheet; (ii) CIB Near Type, if the copy is in close proximity to the model to be copied (< 10 mm); and (iii) no CIB if the copy is restricted to the lower half of the sheet, as per literature [37].

### Statistical analysis

Statistical analysis was performed using SPSS software (IBM SPSS Statistics, version 28.0.1.0). The absolute and relative frequencies of dichotomous and categorical variables were calculated, as well as the mean and standard deviation or median and 25° and 75° percentiles for normally distributed and not normally distributed continuous variables, as appropriate. Incidence of outcome and its 95% confidence intervals (CI) was calculated.

Univariate analysis of baseline variables both by sex and performance at neuropsychological tests was conducted using the analysis of variance for normally distributed continuous variables, the Mann-Whitney test for not normally distributed continuous variables and the Chi square test for dichotomous and categorical variables. We accounted for education and the DAD as possible confounding factors by weighting the data. The sensitivities, specificities, and positive and negative predictive values (PPV and NPV, respectively), as well as areas under the receiver operating characteristic curves (AUROCs) for all considered visuospatial tests were calculated and compared using the paired-sample design option. To determine if visuospatial performance was affected by the degree of disability (DAD), a linear regression model and subsequent diagnostic accuracy analyses were conducted.

Significance level was set at α < 0.05 for all tests.

## Results

Data from 342 patients (mean age 73 years, 58.5% female) were collected. Female patients had lower MMSE scores, were less autonomous (lower IADL), and showed worse performance in memory tests (Table 2). Visuospatial abilities were comparable between men and women, except for the ROCF copying test, which was worse in women (Table 2).

**Table 2:** Univariate analysis of demographic, general cognitive and neuropsychological variables across the entire sample based on sex

Domain	Variables	Men (*n* = 142)	Women (*n* = 200)	*p*
General	Age (yrs), mean (SD)	73 (7)	73 (6)	ns
	Education (yrs), mean (SD)	9.8 (4.5)	7.5 (3.9)	**< 0.0001**
	BADL, median (25°-75°)	0 (0–0)	0 (0–0)	ns
	IADL, mean (SD)	10.6 (3.4)	9.8 (3.5)	**0.028**
	DAD, mean (SD)	78.9 (21.4)	77.0 (21.9)	ns
Cognitive general	MMSE, mean (SD)	24.1 (4.4)	22.2 (5.2)	**0.001**
	MoCA, mean (SD)	20.8 (4.1)	20.2 (1.1)	ns
Short term memory	Corsi-block, median (25°-75°)	3.75 (3.2–4.5)	3.8 (3.3–4.5)	ns
	Numeric digit span, mean (SD)	5.1 (1.4)	5.0 (1.2)	ns
	Word digit span, median (25°-75°)	4.0 (3.3–4.4)	4.1 (3.3–4.5)	ns
Long term memory	Babcock, median (25°-75°)	3.8 (3.2–4.5)	3.2 (0-7.5)	ns
	Rey’s words immediate, median (25°-75°)	27.3 (21.2–33.0)	23.3 (28.7–35.1)	**0.047**
	Rey’s words delayed, median (25°-75°)	4.6 (2.9–6.8)	4.7 (0–7.0)	ns
Attentive function	Matrices test, mean (SD)	31.8 (12.3)	31.8 (12.0)	ns
	TMT-A, median (25°-75°)	99.1 (40.1-136.8)	107.5 (44.5-179.4)	ns
	TMT-B, mean (SD)	319.1 (191.9)	348.7 (230.3)	ns
	TMT-BA, median (25°-75°)	213.4 (113.9-291.1)	217 (103.4–21.7)	ns
Executive function	FAB, mean (SD)	12.1 (4.1)	11.2 (4.6)	ns
Language	Phonemic fluency, mean (SD)	20.3 (10.1)	22.5 (10.3)	0.050
	Category fluency, mean (SD)	16.6 (14.2)	17.4 (14.2)	ns
	TT, median (25°-75°)	32.9 (29.5–36.0)	32.5 (28.9–35.9)	ns
Visuospatial function	ROCF copying, mean (SD)	23.7 (11.2)	20.6 (10.9)	**0.011**
	ROCF recall, mean (SD)	7.9 (5.8)	8 (6)	ns
	Copy of drawings, mean (SD)	10.9 (3.2)	10.3 (3.4)	ns
	Spontaneous clock test, mean (SD)	7.4 (4.5)	6.5 (4.1)	ns
	Copy of clock, mean (SD)	9.2 (4.5)	9.6 (4.4)	ns
	CIB, n (%)	38 (26.8)	48 (24.0)	ns

BADL: Basic Activities of Daily Living; CIB: closing-in behavior; DAD: Disability Assessment for Dementia; FAB: Frontal Assessment Battery; IADL: Instrumental Activities Daily Living; MMSE: Mini-Mental State Examination; MoCA: Montreal Cognitive Assessment; ROCF: Rey-Osterrieth Complex Figure; SD: standard deviation; TMT: Trail Making Test; TT: Token Test

We diagnosed MCI or dementia in 308 patients (90.1%), while 34 were diagnosed with SCD (9.9). According to current clinical criteria, 111 patients were diagnosed with MCI (32.5%), 60 with AD (17.5%), 56 with mixed forms of dementia (16.4%), 43 with vascular dementia (12.6%), 34 with FTD (9.9%) and only 4 with LBD (1.2%). SCD patients were younger and more independent compared to MCI and patients affected by all types of dementia except for those affected by LBD (Table 3).

**Table 3:** Cognitive and functional performances across different diagnosis in the overall sample

Diagnosis	SCD# (*n* = 34)	FTD (*n* = 34)	AD (*n* = 60)	Vascular (*n* = 43)	Mixed (*n* = 56)	LBD (*n* = 4)	MCI (*n* = 111)
Age, mean (SD)	68 (2)	73 (1)*	72 (1)*	75 (1)*	75 (1)*	72 (1)	73 (1)*
MMSE, mean (SD)	27.88 (0.4)	19.6 (1.1)*	19.2 (0.6)*	21.7 (0.7)*	20.9 (0.6)*	25 (1.2)	25.3 (0.3)*
MoCA, mean (SD)	23.7 (0.8)	19.3 (1.5)	18.4 (1.6)	17.8 (1.5)	18 (2)	14.2 (0.1)	20.4 (0.5)
Dementia severity (%)	no dementia	14% questionable 33% mild 29% moderate 24% severe	8% questionable 32% mild 56% moderate 3% severe	23% questionable 33% mild 41% moderate 3% severe	14% questionable 44% mild 40% moderate 2% severe	75% questionable 25% mild	13% no dementia 69% questionable 18% mild
BADL, mean (SD)	0.03 (0.03)	0.62 (0.22)	0.28 (0.1)	0.35 (0.14)	0.61 (0.18)	0.25 (0.25)	0.1 (0.06)
IADL, mean (SD)	12.9 (0.4)	8.0 (0.6)*	9.2 (0.4)*	8.7 (0.5)*	8.3 (0.5)*	9.8 (1.3)	12.1 (0.2)
DAD, mean (SD)	91.6 (3.0)	63.6 (5.3)*	71.5 (2.4)*	69.9 (3.7)*	65.3 (3.2)*	77.5 (10.6)	88.7 (1.2)

^#^ Reference group

*p < 0.05 versus reference group

AD: Alzheimer’s disease; BADL: Basic Activities of Daily Living; DAD: Disability Assessment for Dementia; FTD: frontotemporal dementia; IADL: Instrumental Activities Daily Living; LBD: Lewy body dementia; MCI: mild cognitive impairment; MMSE: Mini-Mental State Examination; MoCA: Montreal Cognitive Assessment; SD: standard deviation; SCD: subjective cognitive decline

In the visuospatial function tests, 211 patients scored below the threshold for the ROCF copying test (61.7%), 49 scored below the threshold in the CD (14.3%), 144 had a poor performance on the CDT (42.1%), and 85 patients showed CIB (24.9%). Patients with poor visuospatial performance were generally severely affected, with lower cognitive and physical performance. Patients with ROCF copying test scores below the threshold were also significantly older. Despite correction for years of schooling, poor visuospatial performance was associated with a lower educational level (Table 4). In 29 patients, 10 of whom were affected by AD, all four tests of visuospatial function were impaired (8.5%).

**Table 4:** Univariate analysis of the demographic, functional and cognitive variables across the entire sample based on the assessment of visuospatial functions

		ROCF	
Variables	Preserved performance	Impaired performance	*p*
Age (yrs), mean (SD)	72 (8)	74 (6)	**0.006**
Education (yrs), mean (SD)	11 (4)	8 (4)	**< 0.001**
MMSE, mean (SD)	25.6 (3.2)	21.8 (5)	**< 0.001**
MoCA, mean (SD)	21.2 (3.9)	18.1 (3.9)	**< 0.001**
BADL, median (25°-75°)	0 (0–0)	0 (0–0)	ns
IADL, mean (SD)	11.5 (3.3)	9.7 (3.3)	**< 0.001**
DAD, mean (SD)	84.3 (19.8)	75.2 (21.8)	**< 0.001**
		**Copy of drawings**	
**Variables**	**Preserved performance**	**Impaired performance**	**p**
Age (yrs), mean (SD)	73 (7)	72 (6)	ns
Education (yrs), mean (SD)	9 (4)	7 (4)	**< 0.001**
MMSE, mean (SD)	24.1 (4.1)	18.4 (5.6)	**< 0.001**
MoCA, mean (SD)	199 (4.1)	18.3 (4.5)	ns
BADL, median (25°-75°)	0 (0–0)	0 (0–0)	**0.046**
IADL, mean (SD)	10.7 (3.3)	8.6 (3.3)	**< 0.001**
DAD, mean (SD)	80.6 (20.6)	69.4 (22.8)	**< 0.001**
		**Clock Drawing Test**	
**Variables**	**Preserved performance**	**Impaired performance**	**p**
Age (yrs), mean (SD)	73 (7)	73 (6)	ns
Education (yrs), mean (SD)	9 (4)	8 (4)	**0.002**
MMSE, mean (SD)	24.9 (3.8)	21.2 (5.3)	**< 0.001**
MoCA, mean (SD)	21.1 (4.0)	19.1 (4.0)	**0.014**
BADL, median (25°-75°)	0 (0–0)	0 (0–0)	ns
IADL, mean (SD)	11.0 (3.3)	9.4 (3.4)	**< 0.001**
DAD, mean (SD)	83.1 (20)	73.8 (22.7)	**< 0.001**
		**CIB**	
**Variables**	**Preserved performance**	**Impaired performance**	**p**
Age (yrs), mean (SD)	73 (7)	73 (6	ns
Education (yrs), mean (SD)	9 (4)	8 (4)	**0.002**
MMSE, mean (SD)	23.9 (4.5)	20.5 (5.3)	**< 0.001**
MoCA, mean (SD)	20.8 (3.7)	18.5 (5.7)	ns
BADL, median (25°-75°)	0 (0–0)	0 (0–0)	ns
IADL, mean (SD)	10.5 (3.5)	9.4 (3)	**0.009**
DAD, mean (SD)	79.9 (21.2)	71.4 (23.2)	**0.003**

BADL: Basic Activities of Daily Living; CIB: closing-in behavior; DAD: Disability Assessment for Dementia; IADL: Instrumental Activities Daily Living; MMSE: Mini-Mental State Examination; MoCA: Montreal Cognitive Assessment; ROCF: Rey-Osterrieth Complex Figure; SD: standard deviation

**Fig. 1:**
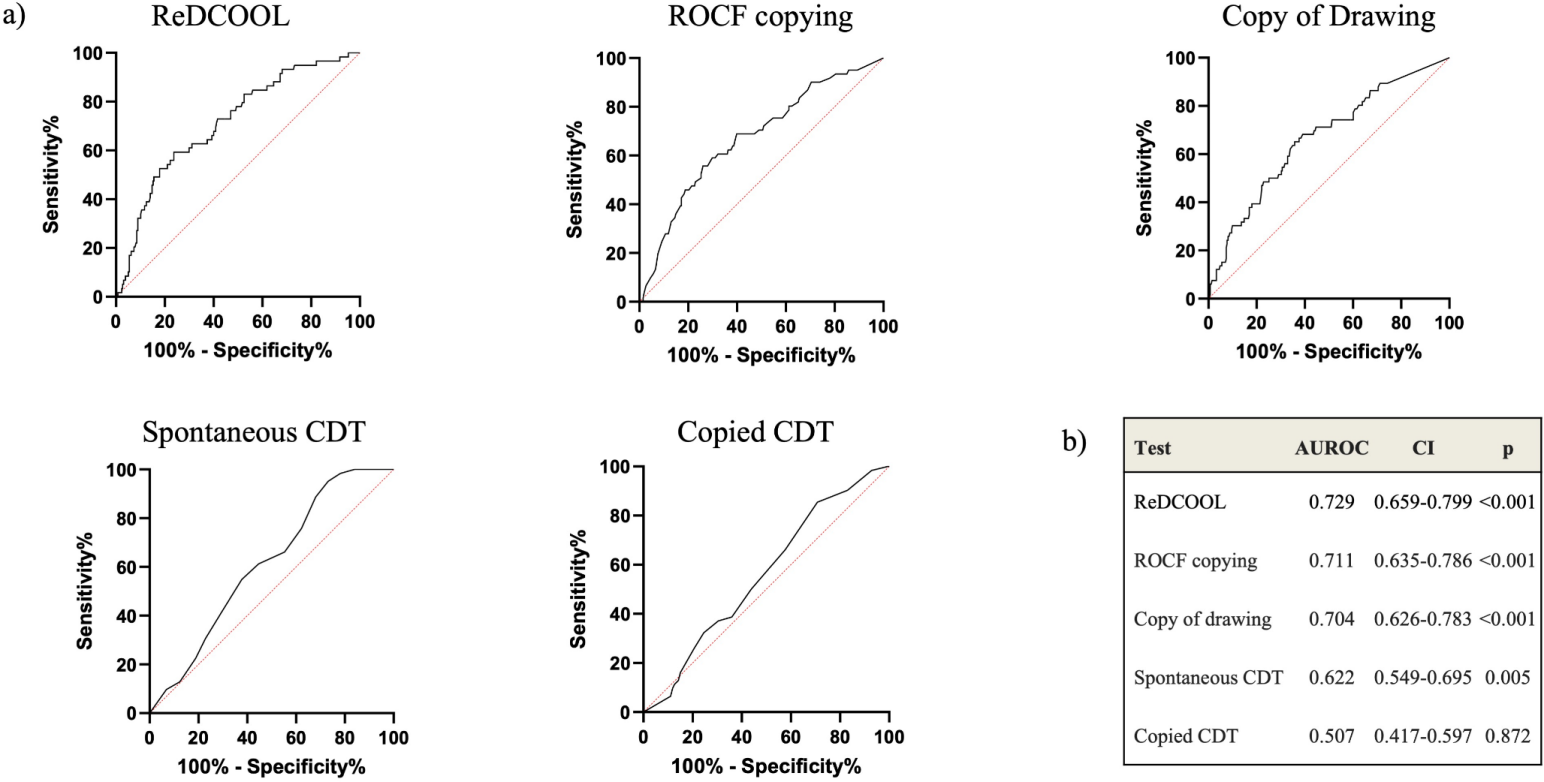
Visuospatial tests performance in AD diagnosis. **A**) ROC curves of different visuospatial tests. **B**) AUROC of different visuospatial tests (CIs and p values are also shown). AUROC: area under the receiving operating curve; CDT: Clock Drawing Test; CIs: confidence intervals; ROC: receiving operating curve

Accuracy measures were calculated for each test in detecting patients affected by AD (Supplementary Table 1). As the performance of the individual tests was not satisfactory in terms of sensibility and specificity, we tested a new index, constructed using the best-performing tests (ROCF copying test, CD, and free-drawn CDT), further corrected for years of education. The additional correction for education was necessary, as differences in years of education were evident in patients with poorer visuospatial performance. Thus, the score was obtained by adding the scores from ROCF, CD, and spontaneous CDT, along with years of schooling (ReDCOOL = ROCF + CD + spontaneous CDT + years spent at school). The ReDCOOL index outperformed the other tests in the differential diagnosis of AD (AUROC 0.729, 95% CI 0.659–0.799; *p* < 0.001) (Fig. 1). There was no significant difference in test performance between sexes. To determine if the level of disability influenced visuospatial performance, data were further adjusted for DAD; however, this correction did not affect ReDCOOL performance.

## Discussion

The primary objective of this study was to determine if a quantitative score, mainly based on a careful evaluation of simple tests assessing visuospatial performance can offer a reliable and cost-effective contribution to enhancing clinicians’ ability to diagnose AD. Here, we demonstrate that using the ReDCOOL index, which combines scores from the ROCF copying test, CD, CDT, and years of education, is superior to each of the visuospatial tests for the clinical diagnosis of AD. Early differentiation between AD and other forms of dementia is crucial for timely appropriate treatment, possibly contributing to slowing of disease progression and to the reduction of the socio-sanitary burden of advanced dementia stages. A comprehensive assessment of visuospatial abilities in AD patients could be useful not only for diagnostic purposes but also for developing rehabilitation strategies to improve functional status in AD.

A primary cause of caregiver stress is the decline in activities of daily living (ADL) and overall functional status of patients [38] and a large amount of evidence shows that visuospatial abilities are major contributors to the everyday functioning of AD patients [39–41].

We observed that, despite being similar in age, women generally exhibited greater cognitive compromise than men in overall cognition, functional status, memory, and visuospatial performance on the ROCF copying test. Differences in education level could contribute to the observed sex differences in visuospatial processing through several mechanisms. Education is known to influence cognitive reserve, which can affect performance on cognitive tasks, including visuospatial abilities. Historically, men and women have had different educational opportunities, which could lead to disparities in cognitive skill development.

Our findings indicate that patients with poorer visuospatial performance exhibited worse cognitive function compared to those with better visuospatial abilities, suggesting a progressive decline in visuospatial performance with dementia progression. Importantly, this decline was not influenced by functional status, as evidenced by the lack of influence of DAD on the performance of the ReDCOOL index. This observation suggests that ReDCOOL may represent a reliable diagnostic tool for AD regardless of functional status.

Several limitations of this retrospective study, conducted on an unselected consecutive sample of patients referred to a single center, need to be acknowledged. The diagnosis was based solely on current clinical criteria without biomarkers or pathological verification. Although imaging information was available for most patients, the imaging data were not systematically collected and had been used only to support the clinical diagnosis. The possible presence of co-pathology (i.e., vascular and degenerative) was based on clinical and imaging criteria but could not be systematically evaluated in all patients. Moreover, while the MMSE was used as a proxy for dementia severity as reported in Literature [42], we acknowledge that it may not fully capture all aspects of disease severity. To address this, we examined whether differences in MMSE scores could account for the observed variations in visuospatial functioning. However, even after adjusting for MMSE scores in our analysis, the differences in visuospatial performance between groups remained significant, suggesting that factors beyond overall cognitive impairment contribute to these findings. Furthermore, the statistical analysis was adjusted for education and level of disability to account for potential confounding factors. However, disease duration is another relevant variable that could influence the outcomes. Due to the unavailability of data on disease duration, we acknowledge this as a study limitation. Nonetheless, previous research [43] suggests that adjustment for the level of disability can serve as a proxy for disease duration, as the degree of impairment often correlates with disease progression. Finally, the application of the score to low-education subjects may be questionable, considering the close link of visuo-spatial abilities with levels of literacy [44]. The calculation of the score requires the administration of the specific tests used for the study. The possibility to use other visuo-spatial processing task needs to be investigated.

On the other hand, this study has several strengths that contribute to its relevance and potential clinical impact. By utilizing a real-world clinical cohort from an outpatient memory clinic, the findings reflect practical diagnostic challenges rather than an artificial research setting. The comprehensive neuropsychological assessment, which includes multiple cognitive domains, places a particular focus on visuospatial function, an area that is often underutilized in the differential diagnosis of AD disease. A key strength is the introduction and validation of the ReDCOOL index, this index demonstrates superior accuracy in identifying AD compared to individual tests. Furthermore, the inclusion of a diverse patient population with various cognitive disorders increases the generalizability of the findings, reinforcing the potential utility of the ReDCOOL index in routine diagnostic workflows.

## Conclusions

In conclusion, we advocate for the use of the ReDCOOL index to enhance confidence in the clinical diagnosis of AD. This score is both easily calculated and time-efficient, as it does not require additional patient assessment beyond standard clinical protocols. By improving diagnostic confidence with a simple, cost-effective, and non-invasive approach, this study contributes to the ongoing search for accessible methods to enhance early AD detection in clinical practice. The authors encourage prospective validation in a larger patient cohort to further establish the clinical utility of ReDCOOL.

## Electronic supplementary material

Below is the link to the electronic supplementary material.

Supplementary Material 1

## Data Availability

All Authors had all access to the data in this work and approved the submission of the present manuscript. All material in this assignment is Authors’ own work and does not involve plagiarism. The data that support the findings of this study are available from the corresponding Author upon reasonable request.
